# Association between Religiosity/Spirituality and Substance Use among Homeless Individuals

**DOI:** 10.1192/j.eurpsy.2024.308

**Published:** 2024-08-27

**Authors:** L. M. Vitorino, P. H. F. Camargo, J. G. Tostes, J. C. L. Ferreira, L. A. G. de Oliveira, J. G. Possetti, M. T. Silva, M. V. C. Guimarães, F. Alckmin-Carvalho, G. Lucchetti

**Affiliations:** ^1^Medicine, Faculty of Medicine of Itajubá; ^2^Medicine, Student at Faculty of Medicine of Itajubá, Itajubá; ^3^Medicine, São Paulo University; ^4^Faculty of Americas, São Paulo, São Paulo; ^5^Medicine, School of Medicine, Federal University of Juiz de Fora, Juiz de Fora, Brazil

## Abstract

**Introduction:**

Alcohol and illicit drug use are highly prevalent among the homeless population. Religiosity and spirituality (RS) have been widely associated with lower substance use. However, evidence of this relationship among the homeless is still scarce.

**Objectives:**

To assess the association between RS and the use of alcohol and illicit drugs among the homeless population of a large Brazilian urban center.

**Methods:**

This cross-sectional study was conducted in São Paulo, Brazil. Aspects such as spirituality (FACIT-Sp12), religiosity (P-DUREL), religious-spiritual coping (Brief-RCOPE), and self-applied questions about current substance use (alcohol and illicit drugs) were evaluated. Adjusted Logistic Regression models were performed.

**Results:**

A total of 456 homeless individuals were included, with an average age of 44.5 (SD=12.6) years. More than half of the participants used alcohol (55.7%) weekly and 34.2% used illicit drugs weekly. The adjusted Logistic Regression models identified that aspects of RS were associated with a lower propensity for alcohol and illicit drug use, whereas negative religious-spiritual coping strategies were associated with a higher propensity for the use of both.

**Conclusions:**

The prevalence of alcohol and illicit drug use among participants was high. Positive RS and religious-spiritual coping were significant protective factors against the use of these substances. Conversely, negative religious-spiritual coping strategies were associated with risk factors.

**Disclosure of Interest:**

None Declared

